# Color polymorphism in organic crystals

**DOI:** 10.1038/s42004-020-0279-0

**Published:** 2020-03-17

**Authors:** Bernardo A. Nogueira, Chiara Castiglioni, Rui Fausto

**Affiliations:** 1 CQC, Department of Chemistry, University of Coimbra, P-3004-535 Coimbra, Portugal; 2 CMIC, Dipartimento di Chimica, Materiali e Ingegneria Chimica “G. Natta”, Politecnico di Milano, Piazza Leonardo da Vinci 32, 20133 Milano, Italy; 3 Department of Chemistry, King Fahd University of Petroleum and Minerals, 31261 Dhahran, Saudi Arabia

**Keywords:** Optical materials, Organic chemistry

## Abstract

Color polymorphism is an interesting property of chemical systems which present crystal polymorphs of different colors. It is a rare phenomenon, with only a few examples reported in the literature hitherto. Nevertheless, systems exhibiting color polymorphism have many potential applications in different domains, such as pigment, sensor, and technology industries. Here, known representative chemical systems showing color polymorphism are reviewed, and the reasons for them to present such property discussed. Also, since some of the concepts related to color polymorphism have been frequently used imprecisely in the scientific literature, this article provides concise, systematic definitions for these concepts.

## Abstract (web summary)

Colour polymorphism is a rare phenomenon that occurs when a compound possesses packing or conformational polymorphs of different colours. Here the authors review representative chemical systems that display colour polymorphism and explore the origins of this property.

## Introduction

This paper focuses on color polymorphism, a fascinating property exhibited by chemical systems that present polymorphs showing different colors. In the first few sections, unambiguous definitions of the structural properties and of the relevant physical concepts related with this subject will be provided, since some of them have been frequently used in an imprecise way in the scientific literature. Then, the physicochemical reasons leading to color polymorphism will be described briefly using an essentially phenomenological approach. The last few sections of this paper will survey representative chemical systems showing color polymorphism.

## Terminology

### Polymorphism

In the crystallographic context, polymorphism, from the Greek poly (many) and morphe (form), also known as crystal polymorphism, refers to the ability of a certain compound to exist in different crystallographic structures, resulting from different packing arrangements of its molecules in the crystal structure^1^. It is worth to differentiate between polymorphism and allotrophism. While the latter term describes the existence of different crystal structures of the same element, polymorphism is used regarding different crystalline structures of compounds. It is also important to mention that in this review, one will not consider solvates, hydrates, or dynamic isomers (including geometric isomers and tautomers) as polymorphic phases, but instead, as pseudopolymorphic states. Hence, a safe criterion for the classification of a system as polymorphic would be if the crystal structures are different but give rise to the same liquid and vapor states.

Why is polymorphism so important? The main reason is that although being composed of the same compound, different polymorphic structures can behave as different materials^2^. Indeed, in general, different polymorphs present different physical, chemical, and mechanical properties. Among others, such properties may include different melting temperature, solubility, vibrational transitions (e.g., different infrared and Raman spectra), electronic transitions (distinct UV–visible spectra), density, surface tension, crystal shape, kinetic stability, crystal hardness, and color. These differences in the properties of crystals of the same compound have important practical implications, and result in a wide range of applications in many domains. Nevertheless, they are not only relevant in the context of the production and commercialization of crystalline materials as pharmaceuticals, agrichemicals, food additives, pigments, and explosives, for example^1^, but they have also been shown to be important from the viewpoint of fundamental research. They help scientists understand some fundamental chemical aspects related to intra- and intermolecular interactions, the driving forces leading to their occurrence, and their structural implications. Polymorphs are also important in the context of intellectual and industrial property, since a new polymorphic structure can sometimes originate new or further legal commercialization protection regarding one compound^3^.

### Conformational polymorphism

Conformational polymorphism can be described as the existence of different conformers of the same molecule in different polymorphic crystal structures^1^. In order to make clear the above statement, it is relevant to clearly define what is a conformer. In simple terms, a new conformation can be defined as the result of a variation of any torsion angle in a molecule. On the other hand, a new conformer only exists if this variation of the torsion angle, implies the appearance of a structure defining a new potential energy well, which corresponds to a different stable structure of the molecule. Therefore, not every pair of conformations are conformers, but only the ones that correspond to distinct minima on the potential energy surface^4^.

Another pair of concepts that are worth to distinguish are conformational arrangement and conformational change. The first one happens always for a flexible molecule in a crystal, even if in a minimal extent. This means that, due to the flexibility of the molecule, the optimal geometry in the gas phase is, in the crystal, slightly modified by the intermolecular interactions in order to minimize the lattice energy. Therefore, conformational arrangements give rise to a new molecular structure, because of the modulation of the intramolecular potential by interactions occurring in the solid state. Instead, conformational changes require a change of conformer, i.e., a change of the molecular structure from one minimum of the intramolecular potential energy surface to another one. This change will result often in a markedly different molecular geometry (different conformer), which will also be adjusted (it relaxes) in the crystal by the intermolecular forces, so that it differs somehow from that corresponding to the geometry of the gas-phase minimum energy structure. Thus, conformational arrangements do not give rise to different conformational polymorphs, while conformational changes do^4^.

Notice, moreover, that intermolecular interactions in the solid state are not merely responsible for molecular structure relaxation, but they can remarkably modify the relative energy of two (or more) different conformations, and eventually stabilize a conformer characterized by a relatively high energy in the gas phase, as indeed it happens in different solvents.

### Packing polymorphism

When two crystalline polymorphs are constituted by a non-flexible molecule or by the same conformer of a flexible molecule, then the type of polymorphism present in the system is designated by packing polymorphism. In this case, the different crystallographic structures arise at the intermolecular level, being determined by different intermolecular interactions that are, in this case, comparatively weaker than the intramolecular interactions defining the adopted conformational geometry.

Non-covalent interactions, acting both at intermolecular and intramolecular levels, include van der Waals interactions (including steric repulsion), hydrogen and halogen bonding, charge-transfer forces, and coulombic interactions, among others^1^. They can be roughly organized into three different categories: (1) nonbonded, non-coulombic (e.g., van der Waals dispersion forces and charge transfer), (2) coulombic, and (3) hydrogen and halogen bonding. The energy range of interactions for group (1) is about 5–20 kJ mol^−1^ (between 5 and 8 kJ mol^−1^ for dispersion interactions and less than 20 kJ mol^−1^ for charge-transfer interactions)^5–8^, while for the hydrogen and halogen bonds, the energy is usually comprised between 4 and 42 kJ mol^−1^. The wide range of energy characteristic of the last type of interactions is due to their strong dependence of the distance and relative orientation between the participating atoms^9^. The long-range coulombic interactions exist on an even wider energy range, depending essentially on the distance between the charged molecules or groups involved^10^.

**Fig. 1:**
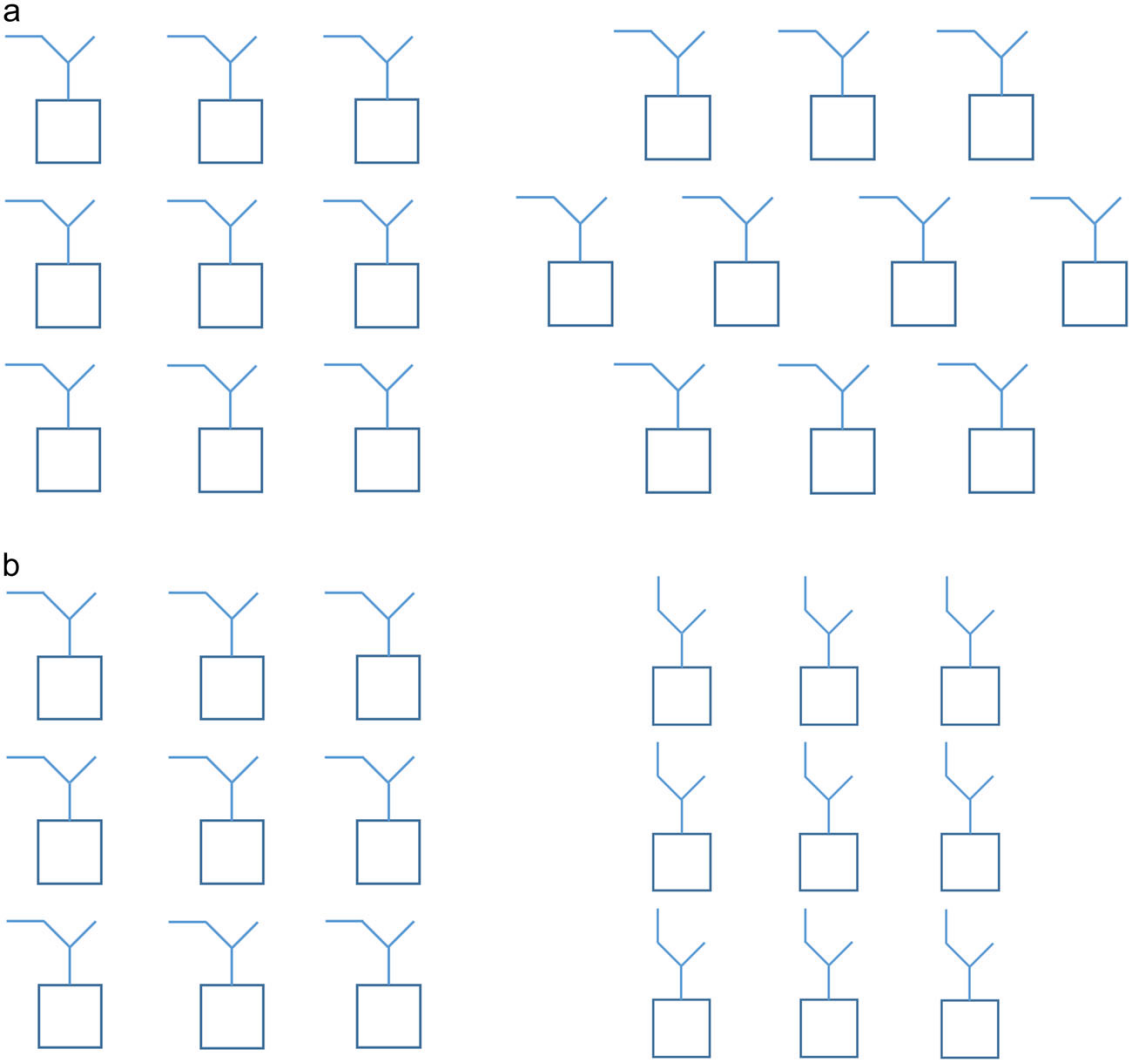
Schematic illustration of the two different classes of polymorphism. **a** Depiction of packing polymorphism and **b** depiction of conformational polymorphism.

The energies associated with intermolecular interactions are, thus, in most of the cases, of the same order of magnitude of those required to distort the molecular geometry in their absence, and are, consequently, likely to affect torsion angles around single bonds, which correspond in general to the most flexible molecular internal coordinates. How much these coordinates are effectively affected by the intermolecular interactions is the key property leading to the two different types of polymorphism, conformational, and packing polymorphism. As one mentioned in the previous section, when intermolecular interactions are strong enough to overcome intramolecular interactions and result in a conformational change, the type of polymorphism displayed is conformational polymorphism. When they are weaker compared with the intramolecular forces acting to stabilize the conformer that is present in the different crystalline structures, one is facing a case of packing polymorphism. Figure 1 schematically represents the difference between the two described types of polymorphism.

### Color polymorphism

It is of fundamental importance to distinguish between color polymorphism and crystallochromism, two terms often mistakenly employed as synonyms. In fact, the correct term to define a molecule that presents polymorphs (both packing and conformational) with different color is color polymorphism. This means that one of the physical properties that varies with the different crystallographic structures of the same compound is, indeed, the color. On the other hand, crystallochromism is correctly defined as the dependence of the solid-state color resulting from small changes in molecular structure specifically related with the length, shape, number, pattern of substitution, and kind of substituent groups^11,12^. This means that different molecules, having the same backbone structure, but different substituents, can present considerably different solid-state colors, despite presenting very similar colors in solution^12^. The misunderstanding between the two definitions appears to have begun in 1994, when Kazmaier and Hoffmann explicitly defined crystallochromism as the dependence of color on crystal packing^13^, a much wider definition where both terms that we are trying to distinguish would fit. It must be stressed that, despite the referred too broad definition, the authors use the term for the correct purpose, as we suggest here. Nevertheless, this wide-ranging definition of crystallochromism leads to the misuse of the term, sometimes appearing as a synonym of color polymorphism^14^, sometimes being used simultaneously for both meanings that are being discussed here^11^. Therefore, and to summarize, color polymorphism should be used when one is dealing with different solid-state colored systems of the same compound, and crystallochromism when referring to crystals that present different colors, but are formed by distinct, yet structurally related compounds (such as compounds belonging to the same family bearing different substitutions on the parent molecule), which present similar colors in solution.

**Fig. 2:**
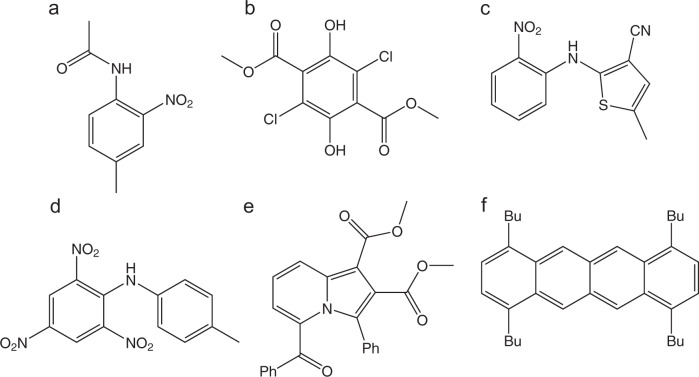
Structures of the parent molecules that give rise to color polymorphs and that are discussed here. **a** N-(4-methyl-2-nitrophenyl)acetamide^20–26^, **b** dimethyl 2,5-dichloro-3,6-dihydroxyterephthalate^27–35^, **c** 5-methyl-2-((2-nitrophenyl)amino)thiophene-3-carbonitrile (ROY)^36–53^, **d** 2,4,6-trinitro-N-(p-tolyl)aniline^54–61^, **e** dimethyl-5-benzoyl-3-phenylindolizine-1,2-dicarboxylate^14^, and **f** 1,4,7,10-tetrabutyltetracene^62–64^. The structures of related compounds that also exhibit color polymorphs are presented in the relevant sections.

Different polymorphs may also exhibit different fluorescence colors, and several fluorescent polymorphic materials whose altered emission between two states under stimuli can be controlled and reversibly switched through interconversion between polymorphs have been reported^15–19^. Despite many potential applications in different areas, such as pigment, sensor, and technology industries, there are only few examples of systems displaying color polymorphism reported in the literature. Specifically, among other technological applications^15^, color polymorphic materials can be used as time–temperature sensors, as described by Cavallini et al.^16,17^, or used in photoelectronic devices with multiplex capabilities, as suggested by Lin et al.^18^. In fact, to the best of our knowledge, there are no more than ten different compound families already synthesized and characterized, and not much more different compounds exhibiting this special type of polymorphism. In this paper, one will focus on the six most representative families of compounds displaying color polymorphism, whose most famous members are represented in Fig. 2: *N*-(4-methyl-2-nitrophenyl)acetamide^20–26^, dimethyl 2,5-dichloro-3,6-dihydroxyterephthalate^27–35^, 5-methyl-2-((2-nitrophenyl)amino)thiophene-3-carbonitrile (ROY)^36–53^, 2,4,6-trinitro-*N*-(*p*-tolyl)aniline^54–61^, dimethyl-5-benzoyl-3-phenylindolizine-1,2-dicarboxylate^14^, and 1,4,7,10-tetrabutyltetracene^62–64^.

## Mechanisms for polymorphs’ color variation

The difference in the color of different polymorphs is caused by a dissimilar electron distribution within the chromophore of their constituting molecules, which affects the electronic energy levels, and consequently, the photons’ frequencies the materials are able to absorb when exposed to light. The distinct electron distributions can be due to the different packing interactions existing in the polymorphs, the different geometry of the conformers present in the crystals, or both.

To understand the change in color induced by a conformational change, one can think of a molecule exhibiting a π-delocalized system in two conformations, where the extension of the delocalization is different. This can be varied by performing an internal rotation about a bond, so that, for example, an initially more planar conformation with an extended π-delocalized system transforms into a less planar conformation where the π-electrons overlapping in the neighborhood of the rotated bond are weakened. In practical terms, the conformational change reduces the length of the π-delocalized system, which leads to increase the energy difference between the excited and the ground electronic states, i.e., to a decrease of the wavelength associated with the electronic absorption responsible for the color of the system. Let us suppose that the molecule, in the initial conformation, absorbs in the green, and that, after the conformational change, it absorbs in the violet. Since one perceives the color of the materials as the complementary color to that associated with the absorption, the initial material would be perceived as being red, while after the conformational change, it would look yellow. A similar reasoning can be applied when the conformational change would work in the opposite way, so that the π-delocalization within the chromophore increases.

The effects on color strictly associated with packing are also easy to conceptualize. A change in the intermolecular potential felt by the molecules might affect the charge distribution, which in turn will result in a change on the spacing of the energy levels. In this case, the direction of the changes is not easy to predict, so that both red and blue shifts may result. Moreover, peculiar intermolecular interactions that may affect the most charge polarization and/or lead to more significant electron charge transfer (e.g., in the presence of strong H bonds), are expected to be the most effective in terms of color changes.

In the next few sections, one will consider the relative importance of the two factors (packing and conformational changes) for the specific systems herein addressed (see Fig. 2). In brief, for *N*-(4-methyl-2-nitrophenyl)acetamide, the chromophore, which in this case is the whole molecule, is sensitive to changes in both the intermolecular interactions and conformational arrangements, generating three different polymorphs of white, yellow, and amber colors; the last two are packing polymorphs, while the first one is a conformational polymorph of the previous ones. A similar situation occurs for 2,5-dichloro-3,6-dihydroxyterephthalate, where yellow, light-yellow, and white crystals appear as a result of a combination of changes in the intermolecular interactions, in particular hydrogen bonding (for white vs. the two yellow polymorphs), and conformation (for all three polymorphs). In the case of ROY, the dissimilarity in the color of the different polymorphs is mainly due to conformational differences, in particular the different values of the dihedral angles defining the relative orientation of the nitrophenyl and thiophene rings (specifically the arrangement about the N–C_(thiophenyl)_ bond). However, these values are themselves determined to a large extent by the different intermolecular forces present in the different crystals, and all the polymorphs of the compound whose crystallographic structure is known are based on only two conformers, with the red and orange polymorphs forming a group, and the yellow polymorphs defining a second group, with only one exception. Within each group, differences in the color shall be characterized as packing color polymorphism, associated with different crystal architectures, where molecules exhibit non-negligible conformational relaxation. The difference in the color of the 2,4,6-trinitro-*N*-(*p*-tolyl)aniline polymorphs is attributed to the different degree of charge delocalization from the amino group lone-electron pair into the aromatic rings. This depends on the strength of the N–H^…^O intramolecular hydrogen bond that is dissimilar in the orange-yellow and dark-red polymorphs of the compound. Since the conformer present in the two polymorphs is the same, this is then a case of packing-induced color polymorphism. For dimethyl-5-benzoyl-3-phenylindolizine-1,2-dicarboxylate, the difference in the color of the orange and the yellow-green crystals results from the different degree of *π*-conjugation between the indozolidine chromophore and the carbonyl groups of its benzoyl substituents, which assume different orientations in the two polymorphs. This is a clear case of conformationally driven color polymorphism. Similarly, the 1,4,7,10-tetrabutyltetracene system exhibits conformational color polymorphism: while in the red crystals the alkyl groups are perpendicularly oriented in relation to the aromatic chromophoric moiety, in the yellow polymorph, the same alkyl groups are essentially coplanar with the chromophore; the distinct alignment of the alkyl chains in the different conformers leads to significantly different electronic distributions throughout the tetracene chromophore, and changes in the crystal packing, giving rise to the color difference.

## Representative chemical systems showing color polymorphism

### *N*-(4-methyl-2-nitrophenyl)acetamide

*N*-(4-methyl-2-nitrophenyl)acetamide (Fig. 2), also known as 4′-methyl-2′-nitroacetanilide, is a compound that exhibits color polymorphism, first reported in 1885, by Gattermann^20^. It is the first known example of a compound presenting this property. Gattermann first identified white and yellow crystals of the compound, while almost a century later, a third form, amber colored, was isolated and structurally characterized by Moore et al.^21^. In 1963, Skulski reported that the white and yellow polymorphs give rise to the same ultraviolet (UV) solution spectrum, but considerably different UV and infrared (IR) solid-state spectra^17^. Based on these later, he proposed that the white polymorph (stable, m.p. 95 °C) should present intermolecular hydrogen bonding, while the yellow polymorph (metastable, m.p. 93.5 °C) should, instead, exhibit intramolecular hydrogen bonds^22^.

This proposal was confirmed in 1983, when Moore, Yeadon, and Palmer solved the crystallographic structure of these two polymorphs^23^. These authors showed that the white crystals (tabular shaped, obtained by recrystallization in aqueous ethanol) are monoclinic (P2_1_/*c* space group) and present strong C = O^…^H–N intermolecular hydrogen bonds between adjacent molecules, which are identical and *c*-glide plane related, forming chains along the *c* direction. In this polymorph, the NH amide bond points toward the opposite direction relatively to the nitro substituent of the aromatic ring. On the other hand, the yellow crystals (filamentary shaped, obtained by light petroleum and carbon disulfide recrystallization) are triclinic (P$\bar 1$), showing two nonequivalent molecules, both exhibiting the NH amide bond pointing toward the nitro substituent with which a N–O^…^H–N intramolecular hydrogen bond is established. The molecules form a columnar-type structure, with each column presenting only one of the two different molecular conformations existing in the crystal.

One year later, the same authors published^16^ the crystal structure of the amber polymorph (needle shaped, obtained by the partial evaporation of a hot solution: petrol containing about 10% v/v of carbon tetrachloride). The crystals are monoclinic (P2_1_/*c*), with the molecules related to a *c*-glide axis and stacked along the *c* direction in a very distinct columnar form. The conformer present in this polymorph is the same present in the yellow form, and does also show a weak N–O^…^H–N intramolecular hydrogen bond.

In 1986, Fletton et al. presented a study revealing the distinctive solid-state IR and the high-resolution solid-state nuclear magnetic resonance (NMR) spectra of the three polymorphs^24^, and more recently Krivoruchka et al.^25,26^ reported on the ability of *N*-(4-methyl-2-nitrophenyl)acetamide to form bifurcated hydrogen bond complexes with several different protophilic solvents (since solvation is not the theme of this paper, the specific structure of these complexes is not described here), thus confirming once again the flexibility of the molecule in relation to H-bonding.

To conclude, the *N*-(4-methyl-2-nitrophenyl)acetamide system is a paradigmatic example where color polymorphism is determined essentially either by packing (between yellow and amber forms) or conformational (between white and the other two forms) polymorphism.

### Dimethyl 2,5-dichloro-3,6-dihydroxyterephthalate

Dimethyl 2,5-dichloro-3,6-dihydroxyterephthalate (Fig. 2), also referred to in the literature as dimethyl 3,6-dichloro-2,5-dihydroxyterephthalate, and its white and yellow polymorphic crystals were first reported by Hantzch, respectively, in 1908 and 1915^27,28^. About 50 years later, Curtin and Byrn^29^ suggested, based on ^35^Cl nuclear quadrupole resonance and IR spectroscopic data, that in the yellow polymorph the molecules should exhibit two C = O^…^H–O intramolecular hydrogen bonds involving the phenolic groups and the carbonyl moiety of the closest located carbomethoxyl substituents, while in the white form the intramolecular H bonds were suggested to be established with the chlorine atoms. They have also reported that a solid-state transformation of the yellow into the white crystals takes place at around 125 °C, and that the melting point of the latter is ca. 185 °C^29^.

In a subsequent investigation, the same authors, alongside Paul, were able to solve the crystallographic structures of the two polymorphs. The yellow crystals (thin plates, obtained from ethanol recrystallization) are triclinic (P$\bar 1$), with one molecule in the unit cell, and exhibit the predicted^24^ strong C = O^…^H–O intramolecular hydrogen bonds. The molecules are practically planar (with the exception of the methyl hydrogen atoms), and are arranged in stacks or columns along the *b* axis. Along the columns, each molecule is placed in such a way that the center of its ring lies almost precisely over the phenolic oxygen atom of the adjacent molecule in the stack. When viewed down the axis through the ring centers, all molecules in a stack show the same sequence of substituents (chloro, hydroxyl, and carbomethoxyl) proceeding clockwise around the ring. The principal intermolecular forces acting in this crystal are van der Waals interactions. The structure of the white crystals (needles, obtained from ether) was also found to confirm the previous structural conclusions extracted from the spectroscopic data, in particular the involvement of the chlorine atoms in intramolecular Cl^…^H–O hydrogen bonds^29^. The crystals are triclinic (P$\bar 1$), and show two molecules in the unit cell, whose planes of the aromatic rings are inclined at an angle of 12.4° with respect to each other. Like the yellow polymorph, the molecules in the crystal of the white polymorph are also arranged in stacks along the *b* axis. However, in this case, for a given stack, the order of attachment of groups around the ring is clockwise in one molecule and counterclockwise in the molecules above and below it (and vice versa). These geometric features allow for the establishment of intermolecular C = O^…^H–O hydrogen bonds between pairs of adjacent molecules, but both the relatively long O^…^H distances and small O^…^H–O angles of these intermolecular hydrogen bonds^30^ are consistent with the participation of the phenolic hydrogen atoms also in an intramolecular hydrogen bond to the neighbor chlorine atoms (thus giving rise to a bifurcated intra-/intermolecular H-bond-type interaction), as suggested by the previously obtained spectroscopic data^30^.

The major structural difference of the molecules of the compound in the yellow and white polymorphs concerns the dihedral angle between the plane of the aromatic ring and the plane of the carbomethoxyl groups (~4° in the yellow crystals and around 70° and 85° in the case of the two nonequivalent molecules present in crystals of the white polymorph)^30,31^. In 1982, Swiatkiewicz and Prasad reported the Raman and the electronic emission spectra of these two polymorphs^32^.

A third, light-yellow polymorph of dimethyl 2,5-dichloro-3,6-dihydroxyterephthalate, has been more recently described by Yang, Richardson, and coworkers^33–35^. The crystals of this polymorph contain centrosymmetric molecules with the carbomethoxyl group, neither coplanar (as in the first reported yellow crystals) nor nearly perpendicular (like in the white form) to the aromatic ring. In the light-yellow crystals, the dihedral angle between the aromatic ring and the carbomethoxyl group is about 40°. As in the case of the yellow polymorph, the molecules in the new light-yellow form show intramolecular C = O^…^H–O hydrogen bonds between the phenolic group and the neighboring carbonyl moiety, and the global molecular packing is also comparable to that of the first reported yellow polymorph. The light-yellow, rounded edged crystals were found to undergo a phase transition into the stable white form at around 100 °C (sometimes they transform at lower temperatures into the yellow polymorph)^28^, and are obtained concomitantly with the other two forms by slow evaporation of the compound from ethanol and ether solutions, being easily isolated by hand separation according to color and morphology.

In conclusion, dimethyl 2,5-dichloro-3,6-dihydroxyterephthalate is an interesting system where, though it clearly shall be defined as a case of conformational color polymorphism, differences in the major intermolecular interactions present in the known polymorphs (for white vs. the two yellow polymorphs), in particular hydrogen bonding, are also very important in structural terms, and in determining the color of the polymorphs.

### 5-Methyl-2-((2-nitrophenyl)amino)thiophene-3-carbonitrile

5-Methyl-2-((2-nitrophenyl)amino)thiophene-3-carbonitrile, or 5-methyl-2-[(2-nitrophenyl) amino]-3-thiophenecarbonitrile (Fig. 2), better known as ROY due to the red, orange, and yellow colors of its polymorphs, is undoubtedly the most famous and the most extensively studied compound that exhibits color polymorphism.

ROY was first reported in 1995 by Stephenson et al.^36^, in a paper where five different polymorphs were identified, and the crystallographic structures of three of them (one red, one orange, and one yellow) were described. These three polymorphs were subsequently studied by two-dimensional ^13^C solid-state NMR (2D-SSNMR) by Smith et al.^37^, in the first published study where 2D-SSNMR was employed to study conformational polymorphism. In the year 2000, Yu et al.^46^ disclosed a sixth polymorph of ROY and presented its crystallographic structure, alongside with those of the two other already-known polymorphs of the compound whose structure had not yet been solved. At that time, ROY was the only compound with more than five reported polymorphic structures in the Cambridge Structural Database (CSD). One year later, Mitchell et al.^47^ published an article about the selective nucleation of ROY polymorphs through epitaxy with single-crystal substrates, reporting also the evidence of a seventh polymorphic form. In 2002, Yu^48^ presented optical-crystallography, single-crystal spectroscopy, and computational chemistry results for all the seven ROY polymorphs known at the time. Three years later, Chen et al.^49^ discovered two new polymorphs of the compound, and reported the crystal structure of one of them.

More recently, a very interesting study also authored by Yu’s group focused on the effects of cross-nucleation during formation of the ROY crystals, and showed that certain polymorphs cannot nucleate without the presence of others^50^. That study also led to the identification of the tenth polymorph of the compound^50^. In 2018, Tan et al.^51^ reported the crystallographic structure of one of the polymorphs that had not been yet solved before, employing X-ray powder diffraction with Crystal Structure Prediction algorithms, increasing to eight, the number of ROY polymorphs present in the CSD. Finally, in the first semester of 2019, Gushurst et al. described the eleventh polymorph (PO13). Its structure was also determined recurring to quantum calculations. Specifically, it was done by matching the experimental powder pattern with one of the previously predicted crystal structures by Vasileiadis et al.^52^, since no single crystal of the PO13 polymorph was obtained^53^.

**Fig. 3:**
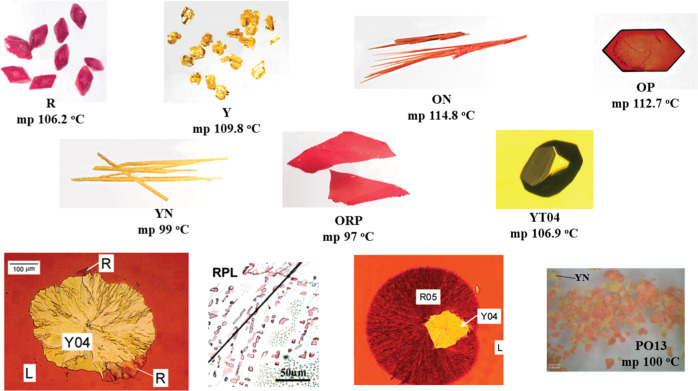
Polymorphs of 5-methyl-2-((2-nitrophenyl)amino)thiophene-3-carbonitrile and their melting point (m.p.). Reprinted (adapted) with permission from Acc. Chem. Res. 2010, 43, 9, 1257–1266. Copyright 2010 American Chemical Society and also from CrystEngComm, 2019, 21, 1363.

With the ninth structure reported, ROY was, once again, at the top of the list of compounds with most polymorphic structures determined, together with flufenamic acid^65^ and aripiprazole^66,67^. However, in the second semester of 2019, two compounds were reported having ten determined crystallographic structures, galunisertib and R-encenicline hydrochloride, overtaking ROY, flufenamic acid, and aripiprazole at the top of the list^68,69^. The 11 different polymorphs of ROY are illustrated in Fig. 3.

The rich polymorphism of ROY results from a combination of favorable thermodynamics and kinetics. The abundance of molecular crystals primarily arises from the conformational flexibility associated with the C–N–C–S torsion angle between the amine and the thiophene moieties, *θ*. The nine different polymorphs of ROY, whose crystallographic structures are known, are designated as R (red prism), Y (yellow prism), ON (orange needle), OP (orange plate), YN (yellow needle), ORP (orange-red plate), YT04 (yellow prism), R05 (red 2005), and PO13 (pumpkin-orange 2013), while the two polymorphs, whose structure is still unknown, are designated by RPL (red plate) and YT04 (yellow prism). The crystallization of the different polymorphs is a rather complex process. Polymorphs R, Y, ON, OP, YN, and ORP were obtained by solution crystallization, and RPL by vapor deposition on single-crystal substrates^38,46,48^. Near room temperature, ROY was found to crystallize spontaneously into YN, Y04, ON, and R05^46,48,49^. YT04 was identified after a solid-solid transition of the metastable polymorph Y04^38,49^, while other several cross-nucleation processes were also described^38,46,50^. Polymorph PO13 was isolated by recrystallization from a supercooled melt of the form YN (previously recrystallized in 2-propanol), at 60 °C^53^.

Every ROY polymorph with solved structure presents one crystallographically independent molecule (*Z*’ = 1) in centrosymmetric packing, and hence, two molecules with mirror-related conformations in the unit cell, with the exception of polymorph RO5 that displays two molecules in the asymmetric unit cell without a center of symmetry. In each crystal polymorph, the ROY molecule adopts a different structure, the largest conformational difference being observed in the torsion angle *θ* (C–N–C–S), which ranges from 21.7° (R) to 122.1° (PO13). It is also interesting to note that the values of this torsion angle roughly correlate with the color of the polymorphs: *θ* = ±21.7, ±39.4, +44.9 (–34.0), ±46.1, and ±52.6^o^ for the red and orange crystals (R, ORP, R05, OP, and ON, respectively), all showing a structure of the ROY molecule, that can be described as the same gas-phase conformer, after a non-negligible structure relaxation driven by intermolecular forces; *θ* = ±104.1, ±104.7, and ±112.8^o^ for the yellow polymorphs (YN, Y, and YT04, respectively), also sharing a common parent conformer, which is different from that associated with the red and orange polymorphs. The above observations suggest that a correlation does exist among the torsional angle values and the trend in colors: values of *θ* closer to 0° correspond to species characterized by light absorption at lower energy, while by increasing the torsional angle, the absorption maximum is blue shifted. Apparently, polymorph PO13 is an exception, since it shows a pumpkin-orange color, but it has the highest torsion angle *θ* value of all the nine structures (122.1°); moreover, it can be considered the result of the relaxation of the same conformer giving rise to the yellow varieties. This apparent discrepancy can be easily justified, considering that the *θ* value of PO13 exceeds 90°, namely the *θ* value characteristic of a hypothetical molecular conformation where the planes of the phenyl- and of the thiophene ring are nearly orthogonal. There are two choices of *θ*, which makes the two rings nearly coplanar: *θ* = 0° and *θ* = 180°. So, if we want to describe effectively the “distortion from planarity” (irrespective of a *trans*- or *cis*-configuration of the two rings), we have to refer to the smaller value between |*θ*| and |*ϕ*| = |180° – *θ*|. Namely, for a torsional *θ* angle greater than 90°, we should consider the corresponding *ϕ* value.

In this way, one can classify the ROY polymorphs in the following way:

– Red polymorphs of ROY, corresponding to |*θ*| = 21.7° (R), 39.4° (ORP), and 44.9° (or 34.0°) (R05).
– Orange polymorphs, with |*θ*| = 46.1° and 52.6^o^ (OP and ON), and |*ϕ*| = 57.9° (PO13).
– Yellow polymorphs, with |*ϕ*| = 75.9° (YN), 75.3° (Y), and 67.2° (YT04).

The above observations clearly indicate the primary factor responsible for the color of the ROY polymorphs. Indeed, the variety of color of ROY polymorphs highlights the relevance of the degree of conjugation between the substituted phenyl and thiophene rings in determining the relative energies of the electronic states of the molecule. A more effective conjugation correlates with an angle between the planes of the two rings approaching to zero, what in fact happens both in the case of |*θ*| (and *r* |*ϕ*|) near 0°. As discussed above, a more extended conjugation leads to a red shift in the visible absorption spectrum, and accordingly, the polymorphs seen as red absorb in the green (the complementary color of red), while those perceived as orange and yellow absorb in the blue and in the violet, respectively.

**Fig. 4:**
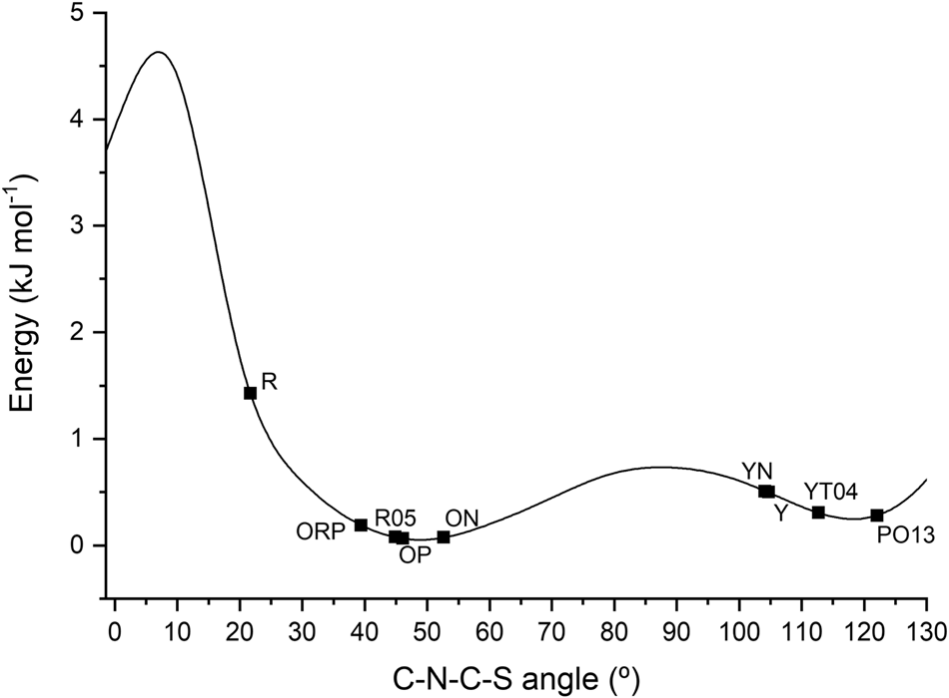
Relative energy as a function of the C–C–N–S torsion angle (*θ*) for the 5-methyl-2-((2-nitrophenyl)amino)thiophene-3-carbonitrile monomer. The values of this torsion angle in the monomers present in each of the polymorphs of the compound are indicated by the solid squares on the energy profile. The calculations were performed at the DFT(B3LYP)/6–311 + G(d,p) level of approximation.

Nonetheless, it shall be stressed that intermolecular forces also play an important role in determining the color of the different polymorphs of ROY. In all polymorphs whose structure is known, the arrangement about the N–C_(phenyl)_ bond of the amino bridge is the same (this corresponds to the geometry shown in Fig. 2), which is justified by the fact that the assumed geometry allows for the establishment of a strong intramolecular hydrogen bond between the amine group and the nitro substituent of the phenyl ring. Since this intramolecular hydrogen bond consumes the best H-bond donor in the molecule, the intermolecular forces presented in ROY polymorphs are essentially of the van der Waals type, which stabilize the different conformations found in the crystals. The relative free energies of seven of the structurally characterized ROY polymorphs have been determined from melting and eutectic melting data obtained by differential scanning calorimetry (DSC), and found to follow the order, over a range of 4 kJ mol^−1^: Y < YT04 < R < OP < ON < YN < ORP^46,49–51^. This order contrasts with the order of the relative energy for the isolated monomeric structures corresponding to the conformations found in the crystals, which is OP < ON < ORP < YT04 < Y < YN < R (Fig. 4), demonstrating the relevance of intermolecular interactions. Different interactions in the different crystal polymorphs are indeed responsible for the displacement of the potential energy minima, namely for the stabilization of torsional angle values different from those of gas-phase equilibrium structures, but they are also responsible for remarkable changes in energy, resulting in a trend totally unpredictable simply considering the isolated molecule.

In conclusion, despite the major importance of the conformational flexibility of the ROY molecule to its extraordinarily rich polymorphism, and though it has been mistakenly said many times, ROY is not just a case of conformational color polymorphism, but also an outstanding case of packing polymorphism. Figure 4 presents the DFT(B3LYP)/6-311+G(2d,p) calculated relative energy of the isolated ROY molecule as a function of the C–N–C–S dihedral angle (line). The angles and specific energy (for the isolated molecule) of each monomeric unit present in the different crystals are represented by the squares. It is clear from this figure that there are only two different conformers (minima at 50° and 120°, respectively) present in the eight polymorphs of ROY, whose crystallographic structure is known. Polymorphs R, ORP, R05, OP, and ON are conformational polymorphs regarding polymorphs YN, Y, YT04, and PO13. On the other hand, these two groups are formed by five and four packing polymorphs, respectively.

**Fig. 5:**
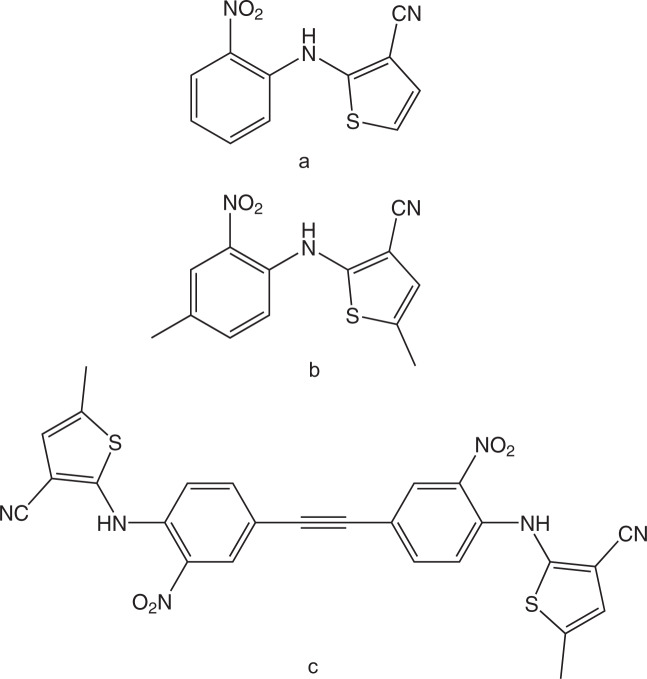
Structures related to 5-methyl-2-((2-nitrophenyl)amino)thiophene-3-6-carbonitrile (ROY). **a** 2-((2-nitrophenyl)amino)thiophene-3-carbonitrile, **b** 5-methyl-2-((4-methyl-2-nitrophenyl)amino)thiophene-3-carbonitrile, and **c** 2,2′((ethyne-1,2-diylbis(2-nitro-4,1-phenylene))bis(azanediyl)) bis(5-methylthiophene-3-carbonitrile).

Three molecules closely related structurally with ROY have also been shown to give rise to color polymorphs, specifically, 2-((2-nitrophenyl)amino)thiophene-3-carbonitrile, 5-methyl-2-((4-methyl-2-nitro-phenyl)amino)thiophene-3-carbonitrile, and 2,2′((ethyne-1,2-diylbis(2-nitro-4,1-phenylene)) bis(azanediyl))bis(5-methylthiophene-3-carbonitrile) (Fig. 5)^39–42^.

2-((2-Nitrophenyl)amino)thiophene-3-carbonitrile (Fig. 5), also known as 2-[(2-nitrophenyl)amino]-3-thiophenecarbonitrile, is a molecule differing from ROY just because it does not possess the methyl substituent in position 5 of the thiophene moiety, studied by Li et al.^42^. Like for ROY, a red (obtained from tetrahydrofuran solution), an orange (obtained from ethanol), and a yellow (only observed during synthesis) polymorph were observed, and the first two characterized structurally. The single-crystal structural data revealed a similar behavior compared with ROY, with the stable red polymorph presenting the thiophene and phenyl rings more coplanar (*θ* closer to 0°) than the metastable orange form.

5-Methyl-2-((4-methyl-2-nitrophenyl)amino)thiophene-3-carbonitrile, or 5-methyl-2-[(4-methyl-2-nitrophenyl)amino]-3-thiophenecarbonitrile (Fig. 5), was synthesized and investigated by He et al.^39^, who found four different polymorphs of the compound (red R, dark red DR, light red LR, and orange O). Although only the DR crystal structure was determined, the thermodynamic relationships between all the polymorphs were determined. Polymorph R was found to be the most stable form above 60 °C, O the most stable form between room temperature and 60 °C, and LR the most stable polymorph below –15 °C, while polymorph DR is metastable throughout the entire studied temperature range. In a subsequent paper from the same group, the solid-solid transformation between the DR and R polymorphs was investigated in detail, allowing to conclude that it is defect driven and accelerated by the presence of water, which can serve as a nucleation catalyst by binding to the crystal surface, especially at defect sites, thus increasing the molecular mobility of these sites and increasing the transformation rate^40^.

Finally, in the case of 2,2′((ethyne-1,2-diylbis(2-nitro-4,1-phenylene))bis(azanediyl))bis(5-methylthiophene-3-carbonitrile) (Fig. 5), also referred to as bis(5-methyl-2-[(2-nitrophenyl)amino]-3-thiophenecarbonitrilyl)acetylene (or simply as el-ROY), which is a dimeric ROY derivative that possesses two chromophores electronically coupled through a triple bond, Lutker, Tolstyka, and Matzger concluded about the existence of at least three different polymorphs^41^. One polymorph (I) was obtained as red needles from evaporation of toluene solution, the second (II) as red-orange plates from dichloromethane, and the third (III) as red-orange rice-shaped needles using polymer-induced heteronucleation. Only the crystal structures of polymorphs I and II were solved, the results showing that, as for ROY and the other ROY derivatives investigated so far, the molecules show the usual intramolecular hydrogen bond between the amino and the nitro groups. The *θ* torsion angles are the smallest ones found hitherto for polymorphs of ROY and ROY derivatives, with the values of 0.1 and 3.3° for polymorphs I and II, respectively^41^. These small angles lead to an almost planar molecular configuration in the crystals, with a high degree of π-conjugation, which is consistent with the red color.

### 2,4,6-Trinitro-*N*-(*p*-tolyl)aniline

2,4,6-Trinitro-*N*-(*p*-tolyl)aniline, or *N*-picryl-*p*-toluidine (Fig. 2), was first reported as a color polymorphic system by Busch and Pungs in 1909, in an article that claimed existence of three different forms: yellow, orange-yellow, and dark-red colored^54^.

The system was further investigated by Cullinane et al.^55^, and by Wood et al.^56^, the X-ray crystallographic structures for the orange-yellow and the dark-red polymorphs being reported in the later study. Yasui et al. ^57^ reported the transformation of the orange-yellow form into the more stable dark-red polymorph through the liquid phase. According to their study, the orange-yellow polymorph melts at 163–164 °C, and then starts to recrystallize into the dark-red polymorphic form, which finally melts at 167 °C. The dark-red form is also the sole one that was possible to obtain by cooling the liquid phase, while the orange-yellow polymorph could only be obtained by recrystallization from solutions^57^. In the more recent study of Braun et al.^58^, the dark-red and orange-yellow polymorphs (together with five different solvates) were investigated by a combined multi-technique approach (single-crystal X-ray diffractometry, hot-stage microscopy, DSC, thermal gravimetric analysis, and IR and Raman spectroscopies). In that work, the dark-red crystals were obtained by slow evaporation of the solvent in different solutions (acetone, acetonitrile, ethyl acetate, methanol, isopropanol, *n*-butanol, and methanol/water), and the orange-yellow form upon recrystallization from acetone/water, chloroform, and mesitylene. The thermal analysis data allow concluding that the dark-red polymorph is the thermodynamically stable form in the entire temperature range investigated (room temperature to 167 °C), as suggested by the earlier study of Yasui et al.^57^. It is also worth noticing that each polymorph was found to sublimate into itself, and the transformation of the orange-yellow form into the dark-red polymorph was only observed in the presence of dark-red seed crystals.

**Fig. 6:**
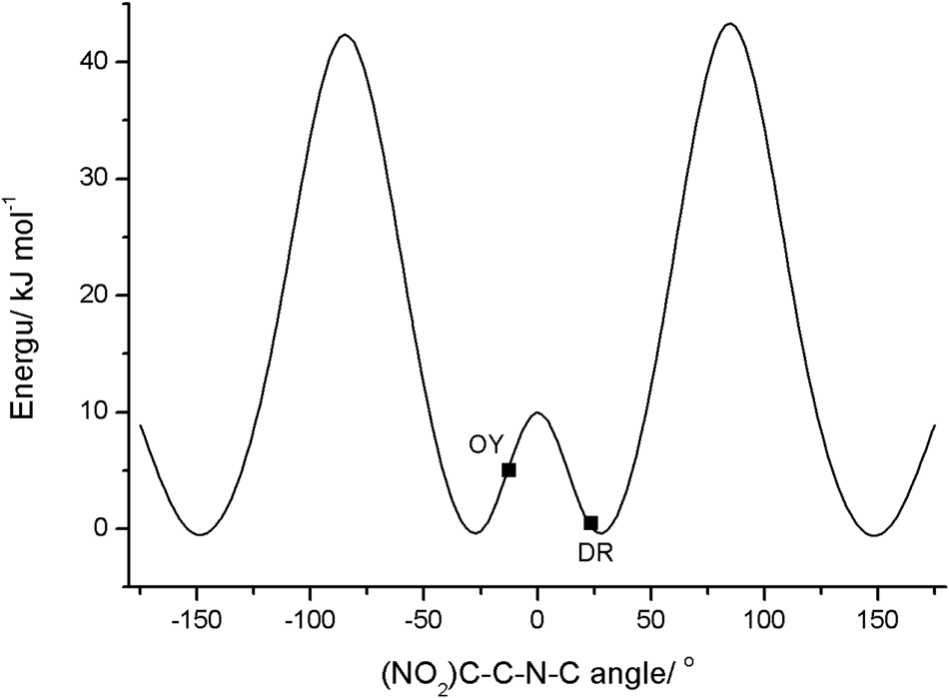
Relative energy as a function of the (NO_2_)C–C–N–C torsion angle for the 2,4,6-trinitro-*N*-(*p*-tolyl)aniline monomer. The values of this torsion angle in the monomers present in both dark-red (DR) and orange-yellow (OY) polymorphs of the compound are indicated by the solid squares on the energy profile. The calculations were performed at the DFT(B3LYP)/6–311 + G(d,p) level of approximation.

Both dark-red and orange-yellow polymorphs crystallize in the monoclinic space group *P*2_1_/*a*, but the values of the two dihedral angles (*τ*_1_ and *τ*_2_) between the aromatic rings are somewhat different in the two polymorphs (23.80° and 43.07°, respectively, for the dark-red polymorph, and –12.52° and –54.35°, respectively, for the orange-yellow form)^58^. In both polymorphs, the molecules adopt a herringbone arrangement, with the neighboring molecules arranged in a head-to-head fashion in the dark-red form and head-to-tail in the orange-yellow one^58,59^. Though the authors classify the two crystalline varieties as conformational polymorphs, this is in fact a case of packing polymorphism. In fact, as it can be seen in Fig. 6, where the potential energy profile for internal rotation about *τ*_1_ [as defined by the (NO_2_)C–C–N–C torsion angle] is represented, the molecule exhibits four minima on the potential energy surface that are equivalent by symmetry, and thus define the same conformer.

In both crystalline structures, an intramolecular N–H^…^O bond between the H atom of the amine and one O atom of the nitro group adjacent to the amine is present. The different colors are attributed to the variation of the length/strength of this H bond, or more specifically, to the associated intramolecular effects, leading to an increased delocalization of the secondary amino nitrogen lone-pair electrons into the aromatic ring moiety. The N–H^…^O intramolecular bond is stronger in the orange-yellow form than in the dark-red polymorph, as the geometrical parameters for the H bond confirm (d(H^…^O) = 1.98 vs. 1.88 Å in the dark-red vs. orange-yellow polymorph).

The two structurally characterized polymorphs have also been studied computationally. Yatsenko and Paseshnichenko demonstrated through their calculations that in the dark-red form, the polarization effect of crystal packing is stronger than the effect of a highly polar solvent, and that under the effect of the crystal electrostatic potential the lowest occupied molecular orbital–highest occupied molecular orbital (LUMO–HOMO) energy gap decreases. In the case of the orange-yellow form, the effect of the crystal field is nearly the same as that of a moderately polar solvent, so that the authors predicted a color-blue shift for this polymorph when compared with the dark-red form, in consonance with the observations, and also in agreement with the packing nature of the observed color polymorphism^60^.

Interestingly, the putative yellow polymorph of the compound has not been described in the literature since the original work of Busch and Pungs^54^. To the best of our knowledge, only the 2001 paper by Ilyina et al.^59^ refers to this species, but the authors suggest that the obtained material might correspond to a water solvate instead of a polymorph of the compound^59^. The precise nature of the yellow phase is still awaiting a definitive answer.

**Fig. 7:**
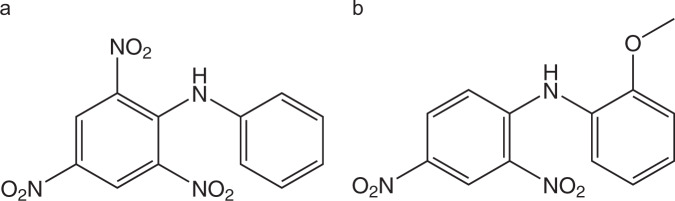
Structures related to 2,4,6-trinitro-*N*-(*p*-tolyl)aniline. **a** Picryl aniline and b) *N*-(2,4-dinitrophenyl)-*o*-anisidine.

There are also some reports on other compounds related to 2,4,6-trinitro-*N*-(*p*-tolyl)aniline, namely on 2,4,6-trinitro-*N*-phenylaniline (also known as picryl aniline)^56^ and *N*-(2-methoxyphenyl)-2,4-dinitroaniline (or *N*-(2,4-dinitrophenyl)-*o*-anisidine)^57,61^ (Fig. 7), but the collected information on these systems is still scarce.

We have also recently started an investigation on this type of compounds. A new 2,4,6-trinitro-*N*-(*p*-tolyl)aniline-related compound with the methyl group bonded in a different position was synthesized and found to exhibit color polymorphism likewise the original compound. The two identified polymorphs have reddish and yellow-orange colors, respectively. Very interestingly, contrarily to what happens for 2,4,6-trinitro-*N*-(*p*-tolyl)aniline, the new compound presents conformational color polymorphism. Also very interestingly, the higher-energy conformer for the isolated molecule gives rise to the most stable polymorph (at room temperature), which is stabilized by the presence of a bifurcated inter- and intramolecular hydrogen bond, while the most stable conformer is present as a constituting unit of the less stable polymorph, which only presents intramolecular H-bonding.

### Dimethyl-5-benzoyl-3-phenylindolizine-1,2-dicarboxylate

Dimethyl-5-benzoyl-3-phenylindolizine-1,2-dicarboxylate (Fig. 2) is a recently synthesized compound that was found to crystallize either as orange cubes (from cyclohexane and hexane/ethyl acetate solutions) or as yellow-green needles (from the same solutions, but with longer crystallization time)^14^.

The yellow-green polymorph crystallizes in the monoclinic space group *C*2/*c*, and the orange polymorphic form crystallizes in the monoclinic space group *P*2_1_/*n*. In both crystal lattices, there are pairs of molecules arranged in an anti-head-to-tail orientation. The bond lengths and angles of the molecules in both structures are almost the same, with one big exception, which is the orientation of the benzoyl group relative to the indolizine part of the molecule. Specifically, the carbonyl bond of the benzoyl group points away from the indolizine core in the yellow-green polymorph, whereas it is aligned in the opposite direction in the orange polymorphic structure. This conformational difference is the main reason of the distinct color of the polymorphs, since it causes a different degree of *π*-conjugation of the carbonyl group with the chromophore (indolizine), which is confirmed by the dissimilar frequencies of the benzoyl C=O stretching vibrational mode in the two crystals^14^. Therefore, dimethyl-5-benzoyl-3-phenylindolizine-1,2-dicarboxylate presents conformational color polymorphism (designated as crystallochromism in the original paper, see Section “Packing polymorphism”).

### 1,4,7,10-tetrabutyltetracene

In 2007, Kitamura et al.^62^ described the synthesis of 1,4,7,10-tetrabutyltetracene (or 1,4,7,10-tetra(*n*-butyl)tetracene), and the discovery of two different color polymorphs of the compound: one red (m.p. 128–130 ^o^C, obtained from recrystallization in chloroform) and one yellow (m.p. 114–116 ^o^C, obtained by recrystallization in *n*-hexane) (Fig. 2). The solid fluorescence spectra of both polymorphs were reported in this study, and are substantially different. In addition, the polymorphic structures were studied by X-ray crystallography. The red polymorph crystallizes in the monoclinic space group *C*2/*c* (*Z* = 4), and the yellow crystals in the triclinic space group P$\bar 1$ (*Z* = 1). The most striking difference between the two polymorphs is the conformation assumed by the alkyl groups. While in the red form the alkyl zigzag chain planes are practically perpendicular to the tetracene backbone, in the yellow polymorph, the alkyl groups are almost coplanar to the tetracene ring. Hence, the molecules in the red polymorph present a chair-shaped structure, while in the yellow form, they are nearly planar. Consequently, being in the presence of distinct conformers in the polymorphs, this system should be classified as a case of conformational color polymorphism, and not as a system displaying packing color polymorphism as it has been suggested in the original work^62^.

Other members of the 1,4,7,10-alkyl-substituted tetracene family were also studied by the Kitamura group, both experimentally and computationally, in particular a series of compounds where the length of the alkyl side chains was varied (from methyl to hexyl derivatives)^63,64^. The solid-state colors of the synthesized tetracenes range through yellow, orange, and red, and depend on the alkyl side-chain length: methyl, propyl, and pentyl derivatives are orange, ethyl is yellow, and hexyl is red (as already mentioned, the butyl-substituted compound exists in two conformational color polymorphs, red and yellow). Structural information for all molecules was obtained, revealing that different alkyl side-chain lengths orient preferentially in different directions, leading to distinct molecular shapes: nearly planar, semi-chair, or chair form, although all the alkyl chains assume an all-*trans*-planar conformation. The packing arrangements for solids of the same color are similar to one another: herringbone-like in the red solids, and slipped-parallel in the orange and yellow solids. With exception of the butyl derivative, described above, only one crystal variety was found for the studied compounds. The mechanism of the observed crystallochromy in the studied series of compounds was discussed by the authors in terms of molecular structure, crystal packing, and calculations that take into account exciton coupling^63,64^.

## Outlook

In this paper, representative chemical systems showing color polymorphism were discussed, and the reasons for them to present such property addressed. Examples were given where the change of conformation is the clearly dominant driven force determining the different color of the polymorphs (as, e.g., in the case of dimethyl-5-benzoyl-3-phenylindolizine-1,2-dicarboxylate and 1,4,7,10-tetrabutyltetracenes). In all these cases, the distinct electron distributions in the polymorphs, leading to color variation, result from subtle modifications in the strengths of specific intramolecular interactions associated with conformational changes. It was noticed that, in these cases, intramolecular hydrogen bonds are efficient determining factors to modulate the geometric characteristics that lead to color changes in the solid state. In other cases, packing is clearly the major cause of the color polymorphism (e.g., as in 2,4,6-trinitro-*N*-(*p*-tolyl)aniline). However, even in these cases, intramolecular bonding appears as a relevant factor in the most striking examples reported hitherto, the packing forces affecting the structures of the molecular units of the crystal in such a way that the H-bonding gains or reduces importance.

Examples where both types of color polymorphism (conformational and packing) are observed, such as in *N*-(4-methyl-2-nitrophenyl)acetamide, dimethyl 2,5-dichloro-3,6-dihydroxyterephthalate, and ROY, have also been discussed, revealing the interplay between the two factors in determining the considered macroscopic property. As a whole, the cited examples, which cover most of the hitherto known chemical systems exhibiting color polymorphism, highlight the general complexity and interrelation of the factors determining this fascinating property.

As in many other cases along the history of science, color polymorphism is nowadays more than just an interesting academic curiosity. Indeed, the applications of color polymorphism in time–temperature sensors and multiplex photoelectronic devices have already given this phenomenon a dimension that goes beyond the academic serendipity. Nevertheless, there is still a world to explore in the field. To design cheap, affordable polymorphic materials that can experience easily controllable color changes, is in fact still an open field of research. Moreover, to use a change of color as a visible indicator of a simultaneous change in other relevant properties of solid materials, for example for magnetic photonic applications, appears also as an avenue to explore. For this, organometallic materials appear a priori as the most promising chemical systems, but investigation on this subject still needs to give the first few steps. Finally, integration of these materials in suitable supports that may make them easier to handle is also a field yet to be developed.
